# Impact of Carbapenem Peri-Transplant Prophylaxis and Risk of Extended-Spectrum Cephalosporin-Resistant Enterobacterales Early Urinary Tract Infection in Kidney Transplant Recipients: A Propensity Score-Matched Analysis

**DOI:** 10.3389/fmed.2022.841293

**Published:** 2022-06-06

**Authors:** Suwadee Aramwittayanukul, Kumthorn Malathum, Surasak Kantachuvesiri, Nuttapon Arpornsujaritkun, Patumsri Chootip, Jackrapong Bruminhent

**Affiliations:** ^1^Department of Medicine, Faculty of Medicine Ramathibodi Hospital, Mahidol University, Bangkok, Thailand; ^2^Division of Infectious Diseases, Department of Medicine, Faculty of Medicine Ramathibodi Hospital, Mahidol University, Bangkok, Thailand; ^3^Division of Nephrology, Department of Medicine, Faculty of Medicine Ramathibodi Hospital, Mahidol University, Bangkok, Thailand; ^4^Ramathibodi Excellence Center for Organ Transplantation, Faculty of Medicine Ramathibodi Hospital, Mahidol University, Bangkok, Thailand; ^5^Vascular and Transplant Unit, Department of Surgery, Faculty of Medicine Ramathibodi Hospital, Mahidol University, Bangkok, Thailand; ^6^Department of Nursing Services, Faculty of Medicine Ramathibodi Hospital, Mahidol University, Bangkok, Thailand

**Keywords:** antibiotic prophylaxis, kidney transplantation, propensity score-matched analysis, extended-spectrum beta-lactamase, pyelonephritis

## Abstract

**Background:**

Urinary tract infection (UTI) is the most common bacterial infection after kidney transplantation (KT), leading to unfavorable clinical and allograft outcomes. Gram-negative uropathogenic bacteria are frequently encountered especially extended-spectrum cephalosporin-resistant (ESC-R) Enterobacterales (EB), causing UTI early after KT.

**Methods:**

A retrospective single transplant study was conducted between January 2016 and December 2019. We performed 1:1 nearest-neighbor propensity score matching without replacement using recipient age, recipient sex, induction, transplant year, human leukocyte antigen, cold ischemia time, and panel-reactive antibody before analyses. Cumulative incidence of ESC-R EB early (within 14 days after KT) UTI was estimated by the Kaplan–Meier method. Risk factors for ESC-R EB early UTI were analyzed by a Cox proportional hazards model. Variables measured after transplantation were considered time-dependent covariates.

**Results:**

We included 620 KT recipients (37% women; mean age ± SD, 43 ± 11 years). Overall, 64% and 76% received deceased-donor allograft and induction therapy. Sixty-five (10%) and 555 (90%) received carbapenems and cefuroxime peri-transplant prophylaxis, respectively. Early UTI occurred in 183 (30%) patients, 52% caused by ESC-R EB. Propensity score matching produced 65 well-balanced pairs. During a 14-day follow-up, the cumulative incidence of ESC-R EB early UTI was 5 and 28% in the carbapenems and cefuroxime groups, respectively (log-rank test = 0.003). Peri-transplant carbapenems prophylaxis was a protective factor against ESC-R EB after KT (hazard ratio, 0.19; 95% confidence interval, 0.05–0.64; *p* = 0.008). Clinical and allograft outcomes did not differ significantly between the groups.

**Conclusions:**

In the setting where ESC-R EB UTI is common among KT recipients, carbapenems peri-transplant prophylaxis could protect against the occurrence of early ESC-R EB UTI after KT. Further prospective studies should focus on this specific infection prevention strategy.

## Introduction

Chronic kidney disease is a significant national public health problem in patients reaching end-stage renal failure. One of the most effective treatments is kidney transplantation (KT) (1). Despite noticeable progress in surgical procedures and immunosuppression after KT, urinary tract infection (UTI) remains an important problem in KT recipients (2–5). UTI is the most frequent infectious complication after KT, occurring in up to 86% of cases (6). Therefore, prevention of UTI must be considered for successful transplantation (7, 8).

The etiological pathogens in UTIs vary depending on environments, hosts' immune status, anatomical structure, virulence factors, or drug susceptibilities. In KT recipients, an emerging multi-drug resistant pathogen, especially Enterobacterales (EB), has been emerging and challenging in clinical practice (9). Uropathogenic *Escherichia coli* (UPEC) is a common bacterial pathogen causing UTIs in KT recipients. Several virulence factors have offered an opportunity to infect vulnerable hosts, mainly, Chaperone-Usher fibers (10, 11). Furthermore, fimbriae have been reported to affect biofilm growth, especially during the early course after KT since KT recipients are indwelled with a urinary catheter and ureteral stent placement (12, 13).

Extended-spectrum cephalosporin-resistant (ESC-R) EB such as *E. coli* and *Klebsiella pneumoniae* were pathogenic for UTI, causing high incidences of infection in KT patients in our setting and internationally (8, 14). Even when antibiotics are used peri-operatively, this pathogen can still survive because it has many mechanisms for antibiotic resistance. However, selecting an appropriate antibiotic is believed to decrease the chance of UTI from this particular bacterium (15). Cefuroxime has been utilized as routine perioperative prophylaxis for KT recipients at our center, although some clinicians sometimes switch to carbapenems due to a concern of an emerging ESC-R EB early UTI after the transplant lately at our institution. In the meantime, using broader spectrum antibiotics could place patients at risk of acquitting multi-drug resistant pathogens is concerning. Therefore, in the present study, we aimed to determine the impact of carbapenems given perioperatively on the incidence of ESC-R EB early UTI among KT recipients whether carbapenems proposed as an appropriate antibiotic that provide an adequate coverage could decrease the rate of ESC-R EB UTI in these vulnerable population.

## Materials and Methods

### Population Study

We included all patients aged ≥18 years who were scheduled to undergo KT at Ramathibodi Hospital between January 2016 and December 2019. The patients were administered intravenous cefuroxime 1.5 g every 8 h or intravenous carbapenems with peri-transplant (first 24 h) prophylaxis started at 30 min before the incision. Patients who received antibiotics other than cefuroxime and carbapenems were excluded from the study. The primary objective was to investigate the effectiveness of carbapenems as prophylactic antibiotics in KT surgery compared with a routine antibiotic (cefuroxime) in preventing ESC-R EB early UTI in KT recipients. The secondary objective was to assess the clinical characteristics, other risk factors, and ESC-R EB early UTI outcomes in KT recipients.

### UTI Definitions

UTI was defined in accordance with the guidelines from the American Society of Transplantation Infectious Diseases Community of Practice 2019 (Supplementary Table S1) (16). Early UTI was defined as UTI that occurred within 14 days after KT. All KT recipients were preemptively screened for UTI after surgery. Patients with asymptomatic bacteriuria were considered to have a UTI in this study because there is no definite recommendation for treatment of these patients and most responsible teams would provide an antibiotic for this condition during the perioperative period. Urine analysis and culture were performed on days 3, 5, 7, 10, and 14 after KT. Only early UTIs caused by *E. coli* and *K. pneumoniae* were evaluated for ESC-R EB. These organisms (including *E. coli* and *K. pneumoniae*) demonstrated resistance to ESC such as ceftriaxone or cefotaxime with a minimum inhibitory concentration (MIC) > 1 mg/L) according to the criteria of the Clinical and Laboratory Standards Institute (17). The Sensititre system (Thermo Fisher Scientific, Oakwood Village, OH) was used as an *in vitro* diagnostic product by the clinical and laboratory standard institute broth microdilution method for clinical susceptibility testing of EB.

The cefuroxime group comprised KT recipients who received cefuroxime for perioperative prophylaxis, while the carbapenem group comprised KT recipients who received meropenem, imipenem, or ertapenem for perioperative prophylaxis.

### Data Collection

The following data were collected: demographic data, comprising sex, age, and etiology of end-stage renal disease; transplant factors, comprising year of transplant, type of allograft, type of immunosuppressants, human leukocyte antigen (HLA) mismatch number, percentage of panel reactive antibody (PRA), donor age and sex, and operation time; UTI data, comprising observed symptoms, type of UTI, pathogens, peri-transplant antibiotic prophylaxis, durations of urinary catheterization and stent after surgery, history of fever in donor, date of diagnosis, and date of initiation and discontinuation of treatment; and outcome data, comprising date of treatment termination, complications such as peri-allograft collection, bacteremia, total length of hospital stay, overall and UTI-related mortality, and allograft function.

### Analyses

Data analyses were carried out using Stata version 12.0 software (StataCorp, College Station, TX) in both whole cohort and propensity score-matched cohort. One-to-one nearest-neighbor propensity score matching using recipient age, recipient sex, induction, transplant year, HLA match, PRA antibody, and cold ischemic time (CIT) without replacement was performed before analyses. The demographic analysis was based on reports of ESC-R EB UTI in patients in a descriptive analysis, and the demographic data were reported as percentage or median. Cumulative incidence of ESC-R EB UTI after KT was estimated by the Kaplan–Meier method. Risk factors for ESC-R EB UTI were analyzed by a Cox proportional hazards model. Variables measured after transplantation were considered time-dependent covariates. *P*-values < 0.05 were considered statistically significant. The study protocol was reviewed and approved by the Institutional Review Board of the Faculty of Medicine at Ramathibodi Hospital, Mahidol University, Bangkok, Thailand (Approval number: ID MURA 2019/806).

## Results

### Baseline Characteristics

**Figure 1:**
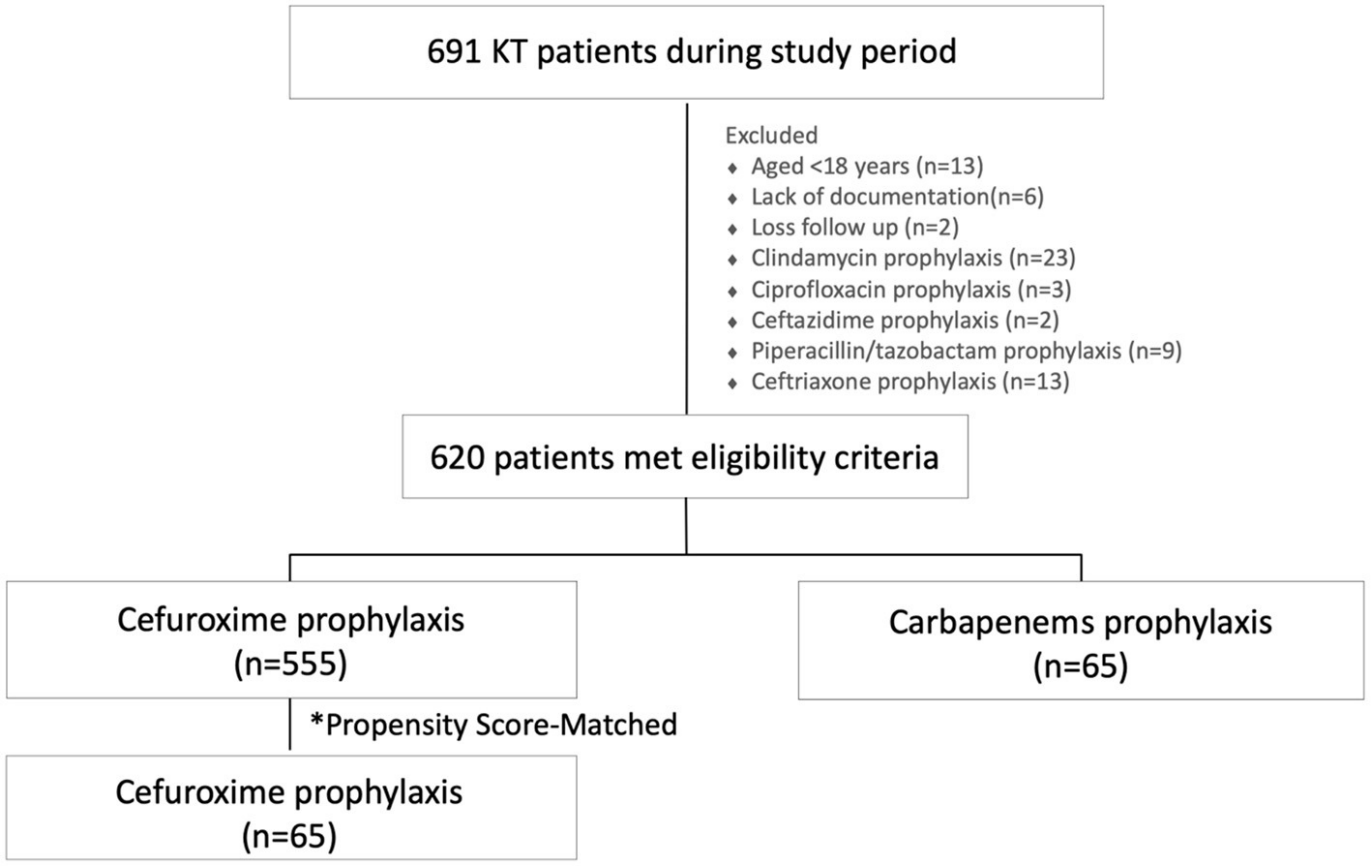
Flow chart of the present study.

A total of 691 KT recipients were identified during the study period (Figure 1), 620 eligible participants were included. Of those, 37 and 63% were women and men with a mean age ± SD of 43 ± 11 years. Overall, 64 and 76% received deceased-donor allograft and induction therapy. Sixty-five (10%) and 555 (90%) patients received carbapenem and cefuroxime peri-transplant prophylaxis, respectively.

After propensity score matching, there were 65 matched pairs between the two antibiotic prophylaxis groups. The baseline characteristics of the matched groups were well-balanced when evaluating standardized biases. Comparisons of the baseline characteristics between the cefuroxime and carbapenem prophylaxis groups before and after propensity score matching are shown in Table 1. The mean recipient age ± SD was 43 ± 11 years, 44% were women, and 56% were men. Most cases of end-stage renal disease occurred for unknown reasons, diabetic nephropathy, or IgA nephropathy. The baseline characteristics after propensity score matching did not differ significantly except for type of KT, because none of the living-related KT recipients received carbapenems for peri-operation prophylaxis.

**Table 1:** Baseline characteristics of the kidney transplant recipients.

**Characteristic**	**Whole cohort**			Propensity score-matched cohort^*^		
	**Cefuroxime** **Group (*n* = 555)**	**Carbapenem** **group (*n* = 65)**	***p*-value**	**Cefuroxime** **group (*n* = 65)**	**Carbapenem** **group (*n* = 65)**	***p*-value**
Recipient age (years), mean (SD)	43 (12)	41 (11)	0.345	43 (12)	41 (11)	0.592
**Recipient sex**			0.091			0.596
Female	197 (36)	30 (46)		27 (42)	30 (46)	
Male	358 (64)	35 (54)		38 (58)	35 (54)	
Induction therapy, *n* (%)	409 (74)	62 (95)	<0.001	62 (95)	62 (95)	>0.999
**Year of KT**, ***n*** **(%)**			<0.001			0.812
2016	145 (26)	10 (15)		14 (22)	10 (15)	
2017	137 (25)	33 (51)		32 (49)	33 (51)	
2018	138 (25)	18 (28)		15 (23)	18 (28)	
2019	135 (24)	4 (6)		4 (6)	4 (6)	
**HLA mismatch groups**, ***n*** **(%)**			<0.001			0.758
0, 1	148 (27)	2 (3)		3 (5)	2 (3)	
2, 3	341 (61)	60 (92)		61 (94)	60 (92)	
4, 5, 6	66 (12)	3 (5)		1 (2)	3 (5)	
CIT (h), median (IQR)	14 (0.5, 18)	17 (15, 21)	<0.001	18 (15, 21)	17 (15, 21)	0.730
**PRA (%)**, ***n*** **(%)**			0.555			1.000
≤50	528 (95)	61 (94)		60 (92)	61 (94)	
>50	27 (5)	4 (6.2)		5 (8)	4 (6)	
**ESKD cause**, ***n*** **(%)**			0.413			0.345
Unknown	366 (66)	38 (58)		36 (55)	38 (58)	
DN	49 (9)	4 (6)		9 (14)	4 (6)	
MN	1 (0.2)	0 (0)		0 (0)	0 (0)	
IgA nephropathy	37 (7)	11 (17)		3 (5)	11 (17)	
IgM nephropathy	1 (0.2)	0 (0)		0 (0)	0 (0)	
MPGN	1 (0.2)	0 (0)		0 (0)	0 (0)	
CGN	26 (5)	1 (1.5)		2 (3)	1 (1.5)	
FSGS	11 (2)	1 (1.5)		3 (5)	1 (1.5)	
LN	19 (3)	4 (6)		5 (8)	4 (6)	
HTN	15 (3)	3 (5)		4 (6)	3 (5)	
Others	30(5.4)	3 (5)		3 (5)	3 (5)	
Donor age (years), mean (SD)	39 (13)	38 (15)	0.459	40 (13)	38 (15)	0.472
Donor female sex, *n* (%)	210 (39)	18 (28)	0.127	17 (26)	18 (28)	0.801
**Type of KT**, ***n*** **(%)**			<0.001			<0.001
LRKT	222 (40)	0 (0)		10 (15)	0 (0)	
DDKT	333 (60)	65 (100)		55 (85)	65 (100)	
Operation time (min), mean (SD)	280 (74)	292 (76)	0.224	280 (60)	292 (76)	0.301
Duration of urinary catheter (days), median (IQR)	6 (5–7)	6 (5–7)	0.971	6 (5–7)	6 (5–7)	0.805
Duration of stent (days), median (IQR)	15 (14–16)	15 (14–16)	0.525	15 (14–16)	15 (14–16)	0.698
ESC-R EB UTI, *n* (%)	94 (17)	3 (5)	0.01	18 (28)	3 (5)	<0.001
**Non-ESC-R EB**, ***n*** **(%)**	460 (83)	62 (95)		47 (72)	62 (95)	
Non-ESC-R EB UTI	74 (13)	11 (17)		7 (11)	11 (17)	
No UTI	386 (70)	51 (78)		40 (61)	51 (78)	

* *Propensity scores were calculated by logistic regression on two groups (carbapenem/cefuroxime) using a set of characteristics including recipient age, recipient sex, induction, transplant year, HLA, CIT, and PRA and grouped into 10 categories for 1:1 matching (carbapenem-to-cefuroxime)*.

*SD, standard deviation; KT, kidney transplantation; HLA, human leukocyte antigen; CIT, cold ischemic time; PRA, panel-reactive antibody; ESKD, end-stage kidney disease; DN, diabetic nephropathy; MN, membranous nephropathy; MPGN, membranoproliferative glomerulonephritis; CGN, chronic glomerulonephritis; FSGS, focal segmental glomerulosclerosis; LN, lupus nephritis; HTN, hypertension; LRKT, living-related kidney transplantation; DDKT, deceased-donor kidney transplantation; IQR, interquartile range; UTI, urinary tract infection; ESC-R EB, extended-spectrum cephalosporin-resistant Enterobacterales*.

### ESC-R EB Early UTI

After KT, early UTI occurred during the follow-up period in 182 (29%) patients in a whole cohort. Those included *E. coli* (*n* = 122), *K. pneumoniae* (*n* = 17), *Pseudomonas aeruginosa* (*n* = 9), *Enterobacter* spp. (*n* = 1), *Enterococcus* spp. (*n* = 19), *Staphylococcus* spp. (*n* = 11), *Streptococcus* spp. (*n* = 1), *Proteus mirabilis* (*n* = 5), *Candida* spp. (*n* = 12), and *Cryptococcus* spp. (*n* = 1). There were 167 (92%) and 15 (8%) patients developed monomicrobial and polymicrobial UTI, respectively. Of the latter, one patient had three isolated organisms. ESC-R EB accounted for 52% of the UTI cases. *E. coli* were susceptible to ertapenem (100%), meropenem (100%), amikacin (99%), piperacillin/tazobactam (92%), cefepime (67%), ceftazidime (63%), ceftriaxone (50%), cefotaxime (49%), amoxicillin/clavulanic acid (76%), trimethoprim/sulfamethoxazole (47%), levofloxacin (44%), and ciprofloxacin (43%). *K. pneumoniae* were susceptible to ertapenem (93%), meropenem (93%), amikacin (97%), piperacillin/tazobactam (79%), cefepime (75%), ceftazidime (67%), ceftriaxone (69%), cefotaxime (65%), amoxicillin/clavulanic acid (68%), trimethoprim/sulfamethoxazole (59%), levofloxacin (69%), and ciprofloxacin (65%).

**Figure 2:**
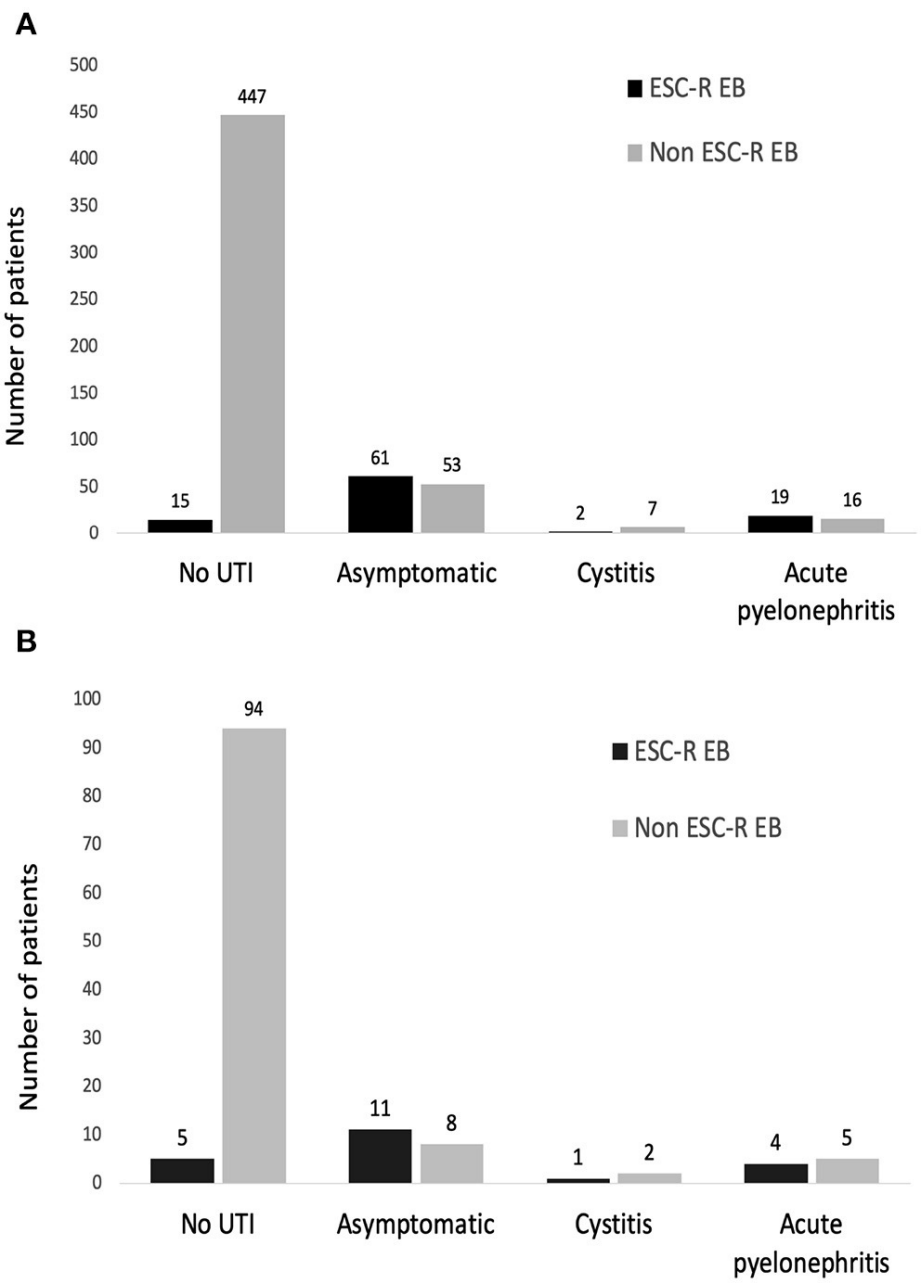
Types of urinary tract infection after kidney transplantation in a whole cohort **(A)** and the propensity score-matched cohort **(B)**.

Of those cases, 74 and 26% % had asymptomatic bacteriuria and symptomatic UTI, respectively. Among those with urinary symptoms, 3% had cystitis, and 23% had acute allograft pyelonephritis (Figure 2A). ESC-R EB accounted for the majority (54%) of the UTI cases. Of these cases, 76% had asymptomatic bacteriuria and 24% had symptomatic UTI (cystitis 5%, pyelonephritis 19%) (Figure 2B).

In the cox proportional hazards model for factors associated with ESC-R EB UTI within 14 days after KT in the whole cohort (Table 2), cefuroxime use at peri-transplant period and female sex were associated with early ESC-R EB UTI in univariate analysis (hazard ratio [HR] 0.19; 95% confidence interval [CI], 0.05–0.75; *p* = 0.019 and HR 3.11; 95%CI, 2.03–4.77; *p* < 0.001). In multivariate analysis, carbapenems and female KT recipients remained independently associated with early ESC-R EB UTI (HR 0.14; 95%CI, 0.04–0.59; *p* = 0.007 and HR 3.24; 95%CI, 2.10–5.00 *p* < 0.001).

**Table 2:** Cox proportional hazards model for factors associated with ESC-R EB UTI within 14 days after kidney transplantation in a whole cohort (*n* = 620).

**Factor**	**ESC-R EB UTI** **group (*n* = 97)**	**Non-ESC-R EB UTI group** **(*n* = 523)**	**Univariate analysis**		**Multivariate analysis**	
			**Hazard ratio (95%CI)**	***p*-value**	**Adjusted hazard** **ratio (95%CI)**	***p*-value**
**Drug**, ***n*** **(%)**						
Cefuroxime	94 (17)	461 (83)	Reference		Reference	
Carbapenems	3 (5)	62 (95)	0.19 (0.05–0.75)	0.019	0.14 (0.04–0.59)	0.007
Recipient age (year)	42 (12)	43 (11)	0.99 (0.97–1.01)	0.123		
**Recipient sex**, ***n*** **(%)**						
Male	38 (10)	355(90)	Reference		Reference	
Female	59(26)	168(74)	3.11 (2.03–4.77)	<0.001	3.24 (2.10–5.00)	<0.001
**ESKD cause**, ***n*** **(%)**						
Unknown	37(17)	179(83)	Reference			
Known	60(15)	344(85)	0.96 (0.62–1.48)	0.857		
Donor age (year), mean (SD)	40(14)	39(14)	1.00 (0.98–1.01)	0.919		
**Donor sex**, ***n*** **(%)**						
Male	59(15)	332(85)	Reference			
Female	38(17)	190(83)	1.12 (0.73–1.72)	0.596		
**History of donor fever**, ***n*** **(%)**						
No	55(17)	267(83)	Reference			
Yes	42(14)	255(86)	0.88 (0.58–1.34)	0.558		
CIT (hours), median (IQR)	14.5 (0.5–19)	15 (0.6–8.5)	1.00 (0.98–1.02)	0.943		
Operation time(min), mean (SD)	281 (68)	281 (76)	1.00 (0.92–1.09) per 30-min increment	0.968		
**HLA mismatch**, ***n*** **(%)**						
0, 1	20 (13)	130(87)	Reference			
2, 3	64(16)	337(84)	1.14 (0.68–1.91)	0.620		
4, 5, 6	13(19)	56(81)	1.09 (0.72–2.03)	0.359		
**PRA**, ***n*** **(%)**						
≤50%	89 (15)	500/ (85)	Reference		Reference	
>50%	8 (26)	23 (74)	1.71 (0.79–3.70)	0.173	1.09 (0.50–2.38)	0.836
**Induction**, ***n*** **(%)**						
No	15 (10)	134 (90)	Reference		Reference	
Yes	82 (17)	389 (83)	1.60 (0.92–2.79)	0.096	1.74 (0.99–3.04)	0.053

*ESC-R EB, extended-spectrum cephalosporin-resistant Enterobacterales; ESKD, end-stage kidney disease; UTI, urinary tract infection; CI, confidence interval; CIT, cold ischemic time; HLA, human leukocyte antigen; LRKT, living-related kidney transplantation; PRA, panel-reactive antibody; DDKT, deceased-donor kidney transplantation; IQR, interquartile range*.

**Figure 3:**
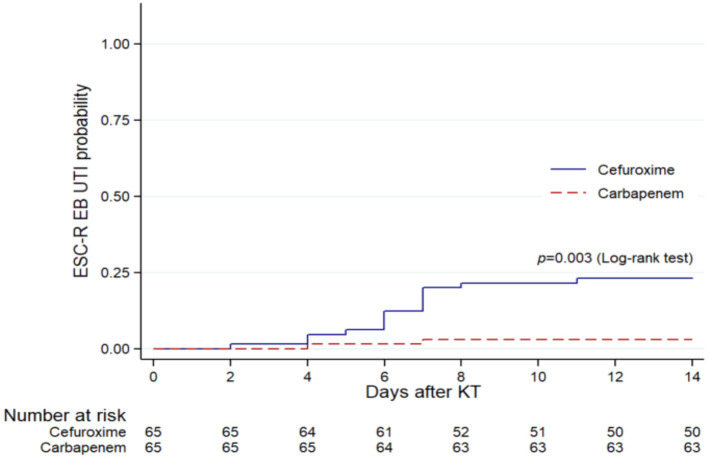
Kaplan–Meier plots of ESC-R EB UTI within 14 days after KT in the cefuroxime and carbapenem groups within the propensity score-matched cohort (*n* = 130).

After propensity score matching, the distributions of pathogens were *E. coli* (*n* = 24), *K. pneumoniae* (*n* = 4), *Pseudomonas aeruginosa* (*n* = 2), *Enterococcus* spp. (*n* = 6), *Staphylococcus* spp. (*n* = 2), *Proteus mirabilis* (*n* = 1), *Candida* spp. (*n* = 1), and *Cryptococcus* spp. (*n* = 1). There were 126 (97%) and 4 (3%) patients who were diagnosed with monomicrobial and polymicrobial UTI, respectively. ESC-R EB accounted for the majority (54%) of the UTI cases. Of these cases, 69% had asymptomatic bacteriuria and 31% had symptomatic UTI (cystitis 7%, pyelonephritis 24%) (Figure 2B). There were 3 (5%) KT recipients in the carbapenem group had ESC-R EB UTI, compared with 18 (28%) KT recipients with the cefuroxime group (*p* < 0.001) (Table 1). The cumulative incidences of ESC-R EB UTI estimated by the Kaplan–Meier method were 0.33% per day (5% per 14 days) in the carbapenem group and 2.20% per day (28% per 14 days) in the cefuroxime group (log-rank test = 0.003) (Figure 3).

In the Cox proportional hazards model, antibiotic prophylaxis was identified as a risk factor significantly associated with ESC-R EB UTI within 14 days after KT. In addition, carbapenems were found to be a protective factor against ESC-R EB UTI compared with cefuroxime (HR, 0.19; 95%CI, 0.05–0.64; *p* = 0.008) (Table 3).

**Table 3:** Cox proportional hazards model for factors associated with ESC-R EB UTI within 14 days after kidney transplantation in the propensity score-matched cohort (*n* = 130).

**Factors *n* (%)**	**ESC-R EB UTI** **group (*n* = 21)**	**Non-ESC-R EB UTI** **group (*n* = 109)**	**Univariate analysis**	
			**Hazard ratio** **(95% CI)**	***p*-value**
**Perioperative prophylaxis**				
Cefuroxime	18 (28)	47 (72)	Reference	
Carbapenems	3 (5)	62 (95)	0.19 (0.05–0.64)	0.008
**History of donor fever**				
No	7 (25)	21 (75)	Reference	
Yes	14 (14)	88 (86)	0.85 (0.31–2.36)	0.757
**Allograft type**				
LRKT	4 (40)	6 (60)	Reference	
DDKT	17 (14)	103 (86)	1.32 (0.25–6.94)	0.742
Operation time (min) (per 30 min)	276 (58)	288 (70)	0.88 (0.69–1.13)	0.318

*ESC-R EB, extended-spectrum cephalosporin-resistant Enterobacterales; UTI, urinary tract infection; CI, confidence interval; LRKT, living-related kidney transplantation; DDKT, deceased-donor kidney transplantation*.

### Outcomes

In the whole cohort (*n* = 620), There were no differences between groups in composite outcomes (such as bacteremia, pyelonephritis, perinephric collection, nephrectomy, slow graft function, delayed graft function, and early allograft dysfunction). However, the duration of antibiotic use and length of hospital stay was significantly longer in the ESC-R EB UTI group.

In a propensity score-matched cohort, there were no differences in composite outcomes between KT recipients who did and did not develop ESC-R EB UTI (Table 4). However, KT recipients who developed ESC-R EB UTI had significantly increased median duration of antibiotic use (8 days; interquartile range, 7–14 days; *p* < 0,001). Furthermore, the rate of bacteremia was significantly higher in patients with ESC-R EB UTI than in patients without (24 vs. 1%, *p* < 0.001). Overall, 1 and 0% developed allograft loss and died, respectively.

**Table 4:** Outcomes of the kidney transplant recipients in the propensity score-matched cohort.

**Factors**	**ESC-R EB group** **(*n* = 21)**	**Non-ESC-R EB** **group (*n* = 109)**	***p*-value**
Duration of antibiotic (days), median (IQR)	8 (7–14)	0 (0–0)	<0.001
Composite complications, n (%)	8 (38)	30 (28)	0.329
Bacteremia	5 (24)	1 (1)	<0.001
Peri-allograft collection	1 (5)	6 (5.5)	>0.999
Recurrent UTI	0 (0)	0 (0)	>0.999
Nephrectomy	0 (0)	1 (1)	>0.999
Delayed allograft function	4 (19)	25 (23)	>0.999
Total length of hospital stays (days), median (IQR)	19 (15–21)	15 (11–23)	0.123
**Hospital mortality**, ***n*** **(%)**	0 (0)	0 (0)	>0.999
Allograft function: GFR (mL/min/1.73 m^2^), mean (SD)			
On day of discharge	58.1 (23.5)	54.7 (20.0)	0.488
At day 30	60.0 (23.2)	57.7 (21.9)	0.666
At day 365	57.5 (22.6)	57.7 (24.6)	0.966

*ESC-R EB, extended-spectrum cephalosporin-resistant Enterobacterales; IQR, interquartile range; GFR, glomerular filtration rate; SD, standard deviation*.

## Discussion

We have reported a propensity score-matched cohort study involving adult KT recipients with similar baseline characteristics in a setting where an ESC-R UTI has been emerging among KT recipients. The results revealed that patients who received carbapenem perioperative prophylaxis had significantly decreased incidence of ESC-R EB UTI within 14 days after KT compared with patients who routinely received cefuroxime perioperative prophylaxis. The incidence of ESC-R EB infection increased the antibiotic duration and likely led to bloodstream infection after transplantation.

UTI is a common complication after KT that was reported to occur in 34–42% of cases (18, 19), similar to the incidence of 30% in the present cohort. A recent retrospective study revealed a significantly high incidence of UTI after KT. Specifically, approximately half of the patients developed UTI within the first month postoperatively, with a median onset of 5 days. Although female sex was identified as an independent factor for UTI (within 1 month after KT) among KT recipients in a single transplant center (20), which is comparable to the results of an analysis in a whole cohort, therefore, we decided to match gender as one of the factors to omit the confounding effect and truly investigate the effect of antibiotic agents. Additionally, KT recipients are subjected to innate immunity impairment, mainly toll-like receptors (TLR). TLR2, TLR4, and TLR5 are essential molecules in innate immunity to defend against pathogens in the genitourinary tract, and calcineurin inhibitors, especially tacrolimus, have been reported to decrease TLR5 expression in bladder macrophages while developing UTI (21, 22). Furthermore, most pathogens were Gram-negative bacilli which lately have been broader resistant to available antibiotics (20). Therefore, comprehensive prevention and treatment measures against known risk factors should be undertaken as early as possible to reduce UTI incidence. Due to a lacking of new effective antibiotics for multi-drug resistant Gram-negative pathogens, the properties of TLRs, as mentioned above, could provide an opportunity for ligand-drug alternatives (23).

A large meta-analysis of KT recipients found an overall rate of 10% for development of extended-spectrum beta-lactamase (ESBL)-producing EB UTI; however, this number could be as high as 33% among Asian KT recipients (24). A retrospective study from a single transplant center in China revealed rate of *E. coli* UTI in KT recipients of 12.5%, of that 73% were identified as having ESBL-producing pathogen and 64% carried adhesions-coding gene (25). In comparison, the incidence of ESC-R EB UTI in our cohort was relatively higher at 16%.

A previous retrospective propensity score-matched study revealed no significant impact of carbapenems compared with any other regimens within the first 72 h in preventing ESBL-producing EB bloodstream infection (26). Our recent study showed that carbapenems did not prevent ESC-R EB bloodstream infection (27). However, ESC-R EB was found to be the leading causative pathogen (50%) for bacteremia within the first year after KT, and 85% of the pathogens were considered to be derived from genitourinary sources (27).

ESC-R EB UTI was presented to be a cause of prolonged antibiotic duration both before and after matched analysis; these data emphasized a significant burden of antimicrobial stewardship, which could conserve patients' microbiome and avoid dysbiosis. However, the hospital stay was longer in the whole cohort, which was not substantially different in the propensity score-matched cohort. Therefore, the length of hospitalization is subjected to multiple factors, including infections and other transplant-related issues. ESC-R EB UTI was found to be associated with high mortality rates in several previous studies (28–30), while our results indicated that it did not increase mortality. Additionally, we also did not observe an increasing rate of carbapenem-resistant Enterobacterales, especially Metallo-ß-Lactamases, in our setting, which could threaten an emerging difficult-to-treat bacterial genitourinary tract infection in these vulnerable populations (31). Although no deaths were recorded during ESC-R EB-related UTI in our cohort, one patient developed allograft loss, and the rate of bacteremia was significantly higher in patients with ESC-R EB UTI, which could place patients at risk of unfavorable outcomes and morbidities.

The main strengths of the present study were the data from a large KT center with a high prevalence of ESC-R EB UTI and the presence of a preemptive urine analysis and culture monitoring protocol. This would have allowed us to retrieve the whole spectrum of UTI, including asymptomatic and symptomatic cases. However, our study had some limitations. The first was its retrospective design, which means that some of the collected patient data in the medical records may have been missing. The second was that the data did not enable us to postulate an effective method against ESBL-producing EB because phenotypic tests were not conducted to confirm ESBL enzyme production. The third was that although a relatively large number of participants were enrolled, the number of participants after matching was limited, which could have prevented us from exploring other risk factors. However, we corrected the unbalanced background characteristics between the two groups to accurately evaluate the effectiveness of carbapenems in preventing ESC-R EB UTI. Finally, the follow-up time was relatively short. Therefore, a prospective study design with a larger number of matched patients together with a more extended follow-up period would yield more statistically significant differences.

Furthermore, the American Society of Transplantation Infectious Diseases Community of Practice recommended using single first-generation cephalosporins such as cefazolin for perioperative prophylaxis in KT recipients (32). Instead, our data propose the potential use of antibiotic prophylaxis based on local epidemiology, predominant with ESC-R EB early UTI, which could lead to post-surgical complications.

In conclusion, ESC-R EB UTI is a potentially serious condition that can arise after KT. Consequently, prevention of this infection should be considered. In a well-balanced retrospective analysis, the present study showed that administration of carbapenem peri-transplant prophylaxis can significantly protect against ESC-R EB UTI early after KT. Appropriate antibiotics coverage during the peri-transplant period could potentially omit infection among KT recipients. Further prospective studies should focus on this particular infection prevention strategy. Furthermore, non-pharmacological interventions such as early urinary prosthesis removal should be encouraged to avoid complicated UTIs from the uropathogenic pathogen.

## Data Availability Statement

The original contributions presented in the study are included in the article/Supplementary Material, further inquiries can be directed to the corresponding author/s.

## Ethics Statement

The studies involving human participants were reviewed and approved by Faculty of Medicine Ramathibodi Hospital, Mahidol University (MURA 2019/806). Written informed consent for participation was not required for this study in accordance with the national legislation and the institutional requirements.

## Author Contributions

JB conceptualized and designed the study. SA, NA, PC, and JB collected the data. SA and JB analyzed data and draft manuscript. All authors reviewed and approved a final version of the manuscript.

## Conflict of Interest

The authors declare that the research was conducted in the absence of any commercial or financial relationships that could be construed as a potential conflict of interest.

## Publisher's Note

All claims expressed in this article are solely those of the authors and do not necessarily represent those of their affiliated organizations, or those of the publisher, the editors and the reviewers. Any product that may be evaluated in this article, or claim that may be made by its manufacturer, is not guaranteed or endorsed by the publisher.
