# Supplementary material for: Impact of Carbapenem Peri-Transplant Prophylaxis and Risk of Extended-Spectrum Cephalosporin-Resistant Enterobacterales Early Urinary Tract Infection in Kidney Transplant Recipients: A Propensity Score-Matched Analysis
Source: Front Med (Lausanne). 2022 Jun 6;9:841293. doi: 10.3389/fmed.2022.841293 (PMC9207318; doi:10.3389/fmed.2022.841293)
Supplement: Supplementary file 1 [file Table_1.docx]

**Supplementary documents**

**Table S1** Definitions of UTI in accordance with the guidelines from the American Society of Transplantation Infectious Diseases Community of Practice 2019. [9]

| Types of UTI | Definitions |
| --- | --- |
| Asymptomatic bacteriuria | No urinary or systemic symptoms of infection combined with >10^5^ CFU/mL uropathogens |
| Acute simple cystitis | Dysuria, urinary urgency/frequency, or suprapubic pain, but no systemic symptoms and no ureteral stent/nephrostomy tube/chronic urinary catheter combined with >10 WBC/mm^3^ and >10^3^ CFU/mL uropathogens |
| Acute pyelonephritis or complicated UTI | Fever, chills, malaise, hemodynamic instability, or leukocytosis (without other apparent etiology); flank/allograft pain; or bacteremia with the same organism as in urine. Dysuria, urgency, frequency, and suprapubic pain may or may not be present together with >10 WBC/mm^3^ and >10^4^ CFU/mL uropathogens |
